# Increased synaptic microtubules and altered synapse development in *Drosophila sec8 *mutants

**DOI:** 10.1186/1741-7007-3-27

**Published:** 2005-12-13

**Authors:** Faith LW Liebl, Kaiyun Chen, Julie Karr, Qi Sheng, David E Featherstone

**Affiliations:** 1 Department of Biological Sciences, University of Illinois at Chicago, 840 W. Taylor St. (M/C 067), Chicago, IL 60607 USA; 2 Department of Cell and Structural Biology, University of Illinois at Urbana-Champaign, 601 South Goodwin Avenue, C626, Urbana, IL 61801 USA

## Abstract

**Background:**

Sec8 is highly expressed in mammalian nervous systems and has been proposed to play a role in several aspects of neural development and function, including neurite outgrowth, calcium-dependent neurotransmitter secretion, trafficking of ionotropic glutamate receptors and regulation of neuronal microtubule assembly. However, these models have never been tested *in vivo*. Nervous system development and function have not been described after mutation of *sec8 *in any organism.

**Results:**

We identified lethal *sec8 *mutants in an unbiased forward genetic screen for mutations causing defects in development of glutamatergic *Drosophila *neuromuscular junctions (NMJs). The *Drosophila *NMJ is genetically malleable and accessible throughout development to electrophysiology and immunocytochemistry, making it ideal for examination of the *sec8 *mutant synaptic phenotype. We developed antibodies to *Drosophila *Sec8 and showed that Sec8 is abundant at the NMJ. In our *sec8 *null mutants, in which the *sec8 *gene is specifically deleted, Sec8 immunoreactivity at the NMJ is eliminated but immunoblots reveal substantial maternal contribution in the rest of the animal. Contrary to the hypothesis that Sec8 is required for neurite outgrowth or synaptic terminal growth, immunocytochemical examination revealed that *sec8 *mutant NMJs developed more branches and presynaptic terminals during larval development, compared to controls. Synaptic electrophysiology showed no evidence that Sec8 is required for basal neurotransmission, though glutamate receptor trafficking was mildly disrupted in *sec8 *mutants. The most dramatic NMJ phenotype in *sec8 *mutants was an increase in synaptic microtubule density, which was approximately doubled compared to controls.

**Conclusion:**

Sec8 is abundant in the *Drosophila *NMJ. Sec8 is required in vivo for regulation of synaptic microtubule formation, and (probably secondarily) regulation of synaptic growth and glutamate receptor trafficking. We did not find any evidence that Sec8 is required for basal neurotransmission.

## Background

The Sec6/8 complex, or 'exocyst', is an octomeric protein complex thought to comprise the proteins Sec3, Sec5, Sec6, Sec8, Sec10, Sec15, Exo70 and Exo84. This complex has been most extensively studied in yeast, where it is required for membrane trafficking and secretion [1,2]. In multicellular organisms, Sec proteins are expressed at particularly high levels in the nervous system [3,4]. The functional role of Sec proteins in neurons, however, including whether they always function as a canonical octomeric complex, remains unclear.

We identified *sec8 *mutants in an unbiased forward genetic screen for mutations causing defects in development of glutamatergic *Drosophila *neuromuscular junctions (NMJs) [5,6]. Sec8 is a core member of the sec 6/8 complex. Alone or as a member of that complex, Sec8 has been suggested to play a role in several aspects of neural development and function, including: (1) neurite outgrowth [7-10], (2) calcium-dependent neurotransmitter secretion [11], (3) trafficking of ionotropic glutamate receptors [12-14] and (4) regulation of neuronal microtubule assembly [10,15].

A mouse *sec8 *knockout has been created, but dies early in embryonic development [16], precluding significant functional or developmental analyses. In contrast, the *Drosophila sec8 *knockouts described here survive through embryogenesis, and hypomorphs survive throughout larval development into pupation. This provides the first opportunity to describe a *sec8 *mutant phenotype at any synapse. The *Drosophila *NMJ is a particularly good model synapse for this purpose, since it is glutamatergic and accessible to powerful microscopic and electrophysiological techniques throughout development. Here, we provide the first description of *sec8 *mutant synaptic phenotypes. We focused our analysis on (1) growth of presynaptic terminals, (2) basal neurotransmission, (3) ionotropic glutamate receptor trafficking and (4) microtubule density, since Sec8 has specifically been implicated in each of these processes.

We found that *sec8 *mutant NMJs show no obvious defect in growth of presynaptic arborizations or neurotransmitter secretion, but do show mild defects in glutamate receptor trafficking and relatively dramatic alterations in synaptic microtubule density. We hypothesize that Sec8's most important role in vivo is spatially-restricted inhibition of microtubule stability, and that disruption of microtubule regulation may underlie most, if not all, *sec8 *mutant phenotypes.

## Results

### Identification and generation of *sec8 *mutants

We identified the transposon insertion mutant *P{SUPor-P}CG2095*^*KG02723 *^in simultaneous unbiased forward screens for *Drosophila *mutants with alterations in NMJ presynaptic growth and glutamate receptor cluster formation [5,6]. We selected this mutant for further study on the basis of a dramatic semi-penetrant presynaptic overgrowth phenotype and reproducible loss of glutamate receptor immunoreactivity (see results, below). The *KG02723 P*-element insertion was mapped by the Berkeley *Drosophila *Genome Project using inverse PCR to the first exon of predicted gene *CG2095 *[17]. We confirmed the KG02723 *P*-element insertion site in *CG2095 *by site-specific PCR primers.

**Figure 1:**
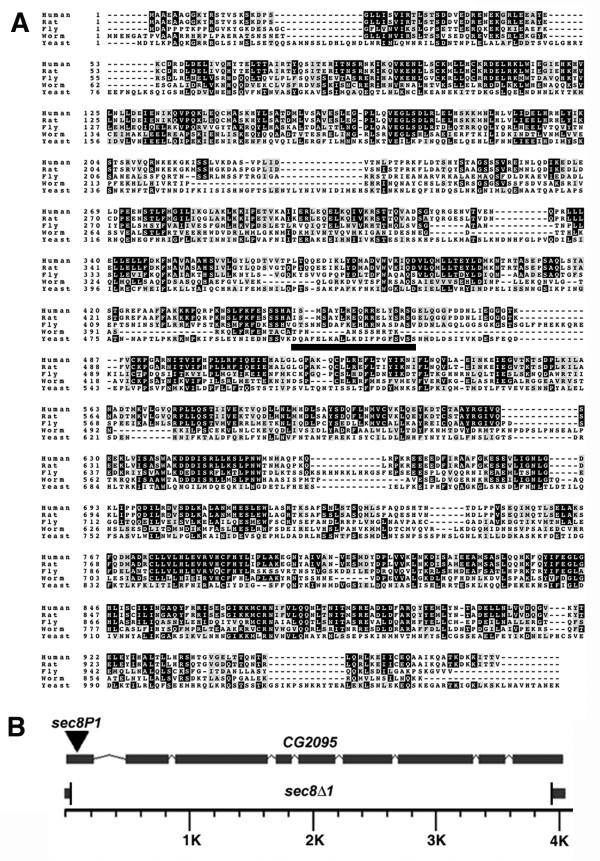
*Drosophila CG2095 / sec8*. A: Amino acid alignment of human (Accession number NP_068579), rat (Accession number Q62824), fly (Accession number AY905551), nematode (Accession number NP_492732) and yeast (Accession number NP_015380) Sec8. The amino acid sequence of *Drosophila *CG2095 is 33% identical to rat and human Sec8. *Drosophila *amino acids 440–460 (underlined) represent the peptide sequence used to generate Sec8 antibodies. B: Genomic map of *Drosophila CG2095 *(*sec8*). Grey bars represent exons. *sec8P1 *is a lethal transposon insertion into the first exon. *sec8Δ1 *is a deletion created by imprecise excision of the transposon. The *sec8Δ1 *deletion removes almost the entire *sec8 *gene, with the exception of the first 37 bp of exon 1 and last 73 bp of the final exon 9.

We cloned and sequenced full-length *CG2095 *cDNA from the wild-type *Drosophila *strain *Oregon R *(Accession number AY905551). A BLAST search using this Oregon R CG2095 amino acid sequence showed 33% identity to rat and human Sec8 (Fig. 1A). Conversely, BLAST searches using mammalian Sec8 against the translated *Drosophila *genome showed close matches only to CG2095. Thus, *CG2095 *represents the sole *Drosophila sec8 *homolog. Hereafter, we refer to *CG2095 *as *sec8*.

Because the *P{SUPor-P}CG2095*^*KG02723 *^P element insertion mutant represents a mutation in the *Drosophila sec8 *gene, we refer to this allele as '*sec8P1*'. We verified that the *P*-element insertion was responsible for the *sec8P1 *phenotype (see below) by complementation analysis and remobilization of the *P*-element [18] to create precise excisions ('revertants'). These revertants are phenotypically indistinguishable from other control (*w*^1118^) animals. Furthermore, *sec8P1 *mutants retain their phenotype in trans to a deficiency, *Df(3R)Tpl10*, that completely eliminates the *CG2095 *gene. These results show that the *sec8P1 *phenotype is specifically due to the P-element insertion in *sec8*.

Many transposon insertion mutants, including the *sec8P1 *mutants, represent hypomorphic alleles. Therefore, we generated deletions of the *sec8 *gene via imprecise excision of the *KG02723 P*-element in *sec8P1*, using standard methods [18]. Among the excision mutants isolated was a *CG2095*-specific deletion that we refer to as '*sec8Δ1*'. To confirm that *sec8Δ1 *only disrupts the *sec8 *gene and provide an accurate molecular description of this mutation, we sequenced the *sec8 *genomic region in *sec8Δ1 *mutants. Sequencing showed that the *sec8Δ1 *deletion begins before the ATG, 37 bp into the first 5' exon, and ends 73 bp prior to the 3' end of the last exon. Neighboring genes are completely intact. *sec8Δ1 *therefore represents a zygotic null. *sec8Δ1 *homozygous mutants hatch at the same time as controls (approximately 24 h after egg laying, AEL), but do not undergo their first molt and die as first instar larvae (~ 46 h AEL). The molecular nature of the *sec8P1 *and *sec8Δ1 *mutations is summarized in Fig. 1B.

Homozygous *sec8P1 *and *sec8Δ1 *mutants both hatched at approximately normal time (22–24 h AEL) and appeared grossly normal by light microscopy (appropriate size, segmentation, mouthooks, etc.). There was no obvious difference in mutant animal size, behavior or viability immediately after hatching. However, homozygous *sec8Δ1 *mutants did not undergo their first molt and did not live past first instar larval stage (~ 48 h AEL). Homozygous *sec8P1 *mutants appeared to molt and pupate normally, and therefore lived through larval development, but did not eclose and therefore died as pupae. In view of the viability and the fact that *sec8 *is almost completely deleted in *sec8Δ1 *mutants, we conclude that *sec8P1 *represents a hypomorphic allele, while *sec8Δ1 *represents a zygotic null allele.

### *Drosophila *Sec8 is abundant in embryonic and larval neuromuscular junctions (NMJs)

**Figure 2:**
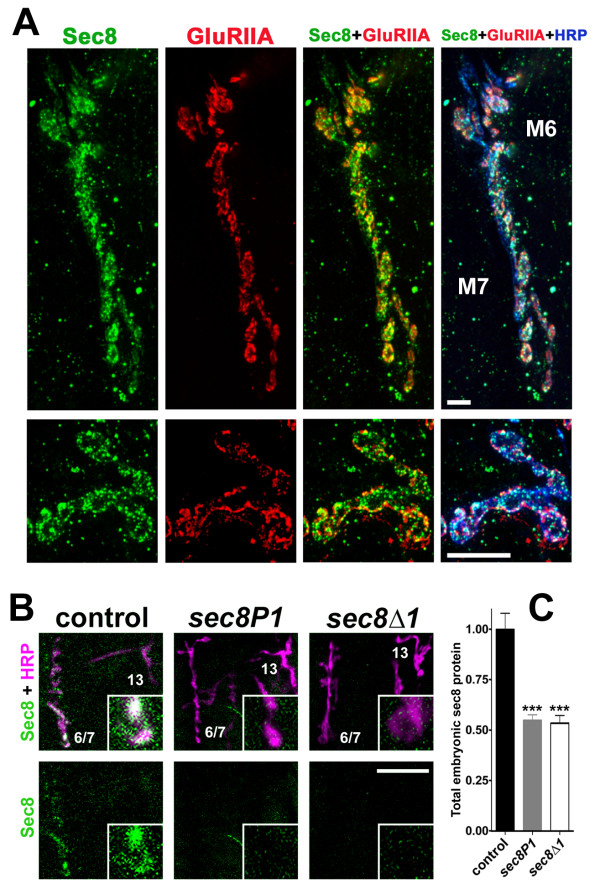
Sec8 is found in the *Drosophila *NMJ. A: Third instar larval NMJs on ventral longitudinal muscles 6 and 7, stained using antibodies against Sec8 (green), GluRIIA (red), and the neuronal membrane marker anti-HRP (blue). All results in this study are derived from experiments on 6/7 NMJs in segments A3–A4. Lower row shows NMJ terminals at higher magnification. Scale bars = 5 μm. B: Confocal fluorescent images showing intersegmental nerve branch b (ISNb) innervating ventral longitudinal muscles in first instar larvae (24–28 h AEL). NMJs were visualized using the neuronal membrane marker anti-HRP (magenta). Sec8 was visualized using anti-Sec8 antibodies (green). Scale bar: 10 μm. Insets show individual synaptic boutons at higher magnification (approximately 2 μm × 2 μm). C: Quantification of total animal Sec8 protein, measured using protein blots from first instar control and mutant larvae, probed with anti-Sec8 antibody.

The *Drosophila *embryonic/larval neuromuscular junction (NMJ) is a well-established model glutamatergic synapse that can be used to examine the effects of Sec8 loss on synaptic development and function. To test whether Sec8 is expressed at the NMJ, we raised rabbit polyclonal antibodies against Sec8 using a synthetic peptide composed of Sec8 amino acids 440–460, and affinity purified these antibodies using the original peptide epitope. Immunoreactivity of this antibody was widely distributed throughout many tissues and was particularly enriched in synapses, including the NMJ (Fig. 2A). Because the pre and postsynaptic membranes are separated by only approximately 15 nm [19,20], it is impossible to distinguish definitively by light microscopy whether Sec8 immunoreactivity directly at the NMJ is pre or postsynaptic. Nevertheless, several indications suggest that Sec8 is both pre and postsynaptic. First, there is clearly Sec8 immunoreactivity throughout the muscle cell outside the area delimited by presynaptic HRP staining; therefore, sec8 must be expressed in the postsynaptic muscle cell. Postsynaptic localization of Sec8 is also suggested by the fact that Sec8 immunoreactivity overlaps immunoreactivity for the postsynaptic glutamate receptor subunit GluRIIA. Three-dimensional reconstructions and rotations (not shown) show Sec8 immunoreactivity extending above the interior face of the muscle, consistent with presynaptic localization. We did not observe any Sec8 immunoreactivity in segmental nerve axons, suggesting that presynaptic Sec8, if present, must be localized specifically at the terminals, but there is little or no immunoreactivity in the centers of presynaptic boutons. Thus, presynaptic Sec8 must be localized specifically near the terminal membrane. We obtained essentially identical results using a recently described and independently generated antibody [21] (data not shown).

Sec8 antibody immunoreactivity in the embryonic/first instar (L1) neuromusculature was dramatically reduced in homozygous *sec8P1 *mutants, and was almost undetectable in *sec8Δ1 *mutants (Fig. 2B). On immunoblots using whole-animal extracts, our Sec8 antibody recognized a single 111 kDa band (not shown). The intensity of this band was reduced, on average, to approximately one-half normal in both *sec8P1 *and *sec8Δ1 *L1 mutants (Fig. 2C). This suggests that despite the deletion of the *sec8 *gene in *sec8Δ1 *mutants, and almost complete elimination of Sec8 in the NMJ of homozygous mutants, there is substantial maternal contribution of Sec8 in other tissues, as previously shown for other sec proteins [7,8,21-23].

We conclude from our immunohistochemical results that Sec8 is probably expressed both pre and postsynaptically at the *Drosophila *NMJ, and that *sec8 *mutant NMJs have almost no remaining Sec8 but substantial maternal Sec8 persists in other parts of the *sec8Δ1 *mutant animals.

### *sec8 *mutants show increased NMJ growth during larval development

Sec proteins have been implicated in the membrane addition required for neurite outgrowth [7-10]. Formation of *Drosophila *larval NMJs involves extensive neurite outgrowth and presynaptic arborization. Examination of motor nerve terminals in the ventral body wall neuromusculature of *Drosophila sec8 *mutants therefore provides an excellent opportunity to test whether Sec8 plays a role in nerve terminal growth in vivo. Approximately halfway through *Drosophila *embryonic development (12 h After Egg Laying, AEL), motor neuron growth cones exit the CNS and travel along the developing segmental nerve toward their body wall muscle targets. Approximately 13 h AEL, development of ventral NMJs begins when motor neuron growth cone filopodia contact the target muscles. Over the next several hours, filopodia-ringed growth cones in contact with appropriate targets collapse to form nascent presynaptic processes of indistinct shape, termed 'prevaricosities'. By 17–22 h AEL, parts of the prevaricosities begin to constrict and/or swell such that presynaptic terminals (known as synaptic boutons) are formed [24-30]. These presynaptic boutons are functional very early; electrophysiological analysis shows that NMJ transmission occurs within minutes of contact between motor nerve axons and muscles [31,32]. At the time of hatching (22–24 h AEL), presynaptic boutons are still relatively indistinct, morphologically, but nonetheless are clearly highly functional since neuromuscular transmission is required for hatching and subsequent crawling of the newly hatched first instar (L1) larva. During larval development (24–120 h AEL), the presynaptic arborization grows dramatically in order to accommodate rapidly growing larval muscles. This growth involves additions in NMJ length, branches and bouton number (from about 8–10 in at hatching to 50–100 in the third instar 6/7 NMJ). Boutons also become more distinct during NMJ development, such that the third instar NMJ typically appears as 'beads on a string' [28-30,33].

**Figure 3:**
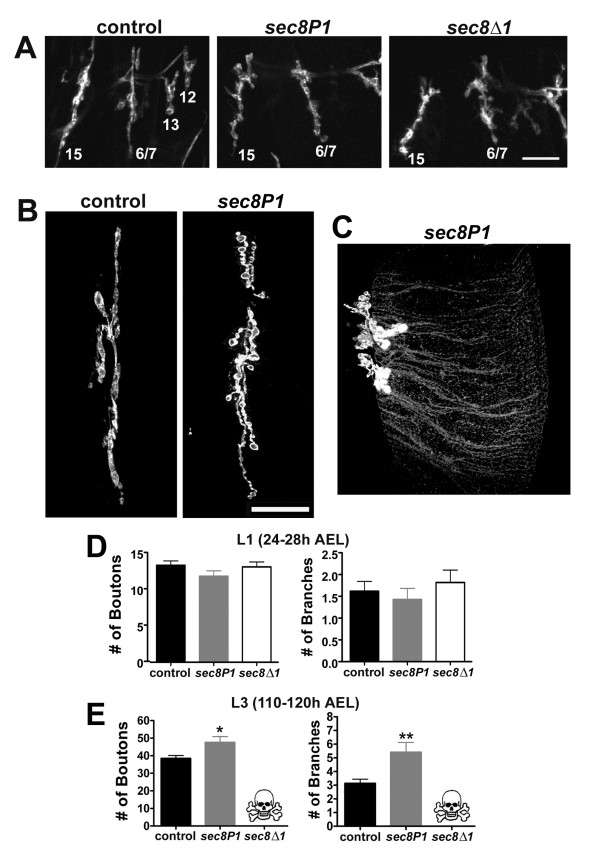
NMJ morphology in *sec8 *mutants becomes abnormal during larval growth. A: Confocal images showing NMJs on ventral longitudinal muscles 15, 6/7, 13 and 12 in first instar larvae (24–28 h AEL), visualized using anti-HRP antibodies. Scale bar: 10 μm. B: Confocal images showing NMJs on ventral longitudinal muscles 6 & 7 in third instar larvae (110–120 hr AEL), visualized using anti-HRP antibodies. Scale bar: 20 μm. C: Example of extreme NMJ overgrowth occasionally observed in *sec8P1 *mutant third instar larvae. The mutant NMJ appears contracted, with an abnormally high number of boutons, compared to controls (c.f. 3B). In addition, long slender neurites (normally not present) extend across the muscle to the right (distally) of the NMJ center. Abnormal NMJ growth such as this was never observed in control animals (N>200). D: Quantification of the number of presynaptic boutons (left) and the number of branches (right) at the 6/7 NMJ in first instar (24–48 h AEL) larvae. E: Quantification of the number of presynaptic boutons (left) and the number of branches (right) at the 6/7 NMJ in third instar (110–120 AEL) larvae. *sec8Δ1 *mutants die before third instar (skull and crossbones) and thus third instar morphology could not be assayed in that genotype.

To examine motor nerve terminal morphology, we used fluorescently-conjugated anti-HRP antibodies (which allow visualization of all neuronal membrane), and confocal microscopy (Fig. 3). In *sec8P1 *and *sec8Δ1 *mutants, NMJ morphology appeared normal in first instar (L1) larvae (Fig. 3A). L1 mutant motor nerve insertion sites appeared normal, presynaptic swelling size was appropriate for the animal age, and the arborizations were similar in number and shape to those from age-matched control animals. The number of boutons and branches in mutant L1 ventral longitudinal muscle 6/7 NMJs were statistically unchanged compared to control animals (Fig. 3D; Control boutons per 6/7 NMJ = 13.36 ± 0.62, n = 14; *sec8P1 *= 11.85 ± 0.75, n = 13, p = 0.130; *sec8Δ1 *= 13.14 ± 0.67, n = 14, p = 0.816; control branches per 6/7 NMJ = 1.62 ± 0.22, n = 21; *sec8P1 *= 1.43 ± 0.25, n = 21, p = 0.577; *sec8Δ1 *= 1.82 ± 0.28, n = 22, p = 0.587). However, in *sec8P1 *third instar larvae (*sec8Δ1 *mutants did not survive to this stage of development), muscle 6/7 NMJs showed consistent and qualitatively obvious changes in morphology and terminal number (Fig. 3B). With less penetrance, *sec8P1 *mutant third instar larvae showed dramatically abnormal neurite growth (e.g. Fig. 3C) that was never observed in control NMJs. The average number of presynaptic boutons and branches was significantly increased in *sec8P1 *mutant larvae compared to controls (Fig. 3E; Control boutons per 6/7 NMJ = 38.45 ± 1.65, n = 22; *sec8P1 *= 47.50 ± 3.36 n = 22, p = 0.020; control branches per 6/7 NMJ = 3.14 ± 0.30, n = 22; *sec8P1 *= 5.41 ± 0.71, n = 22, p = 0.006). Homozygous *sec8P1 *and *sec8Δ1 *L1 mutants did not show any obvious defects in body wall muscle shape or size. Muscle insertions were correct and muscles did not detach abnormally during manual dissection or subsequent treatment. We conclude from these results that loss of Sec8 does not significantly impair growth of motor nerve terminals or presynaptic arborization. Instead, surprisingly, loss of Sec8 triggered an increase of NMJ size during larval development.

### *sec8 *mutants show robust neurotransmission but a slight decrease in the number of postsynaptic glutamate receptors

**Figure 4:**
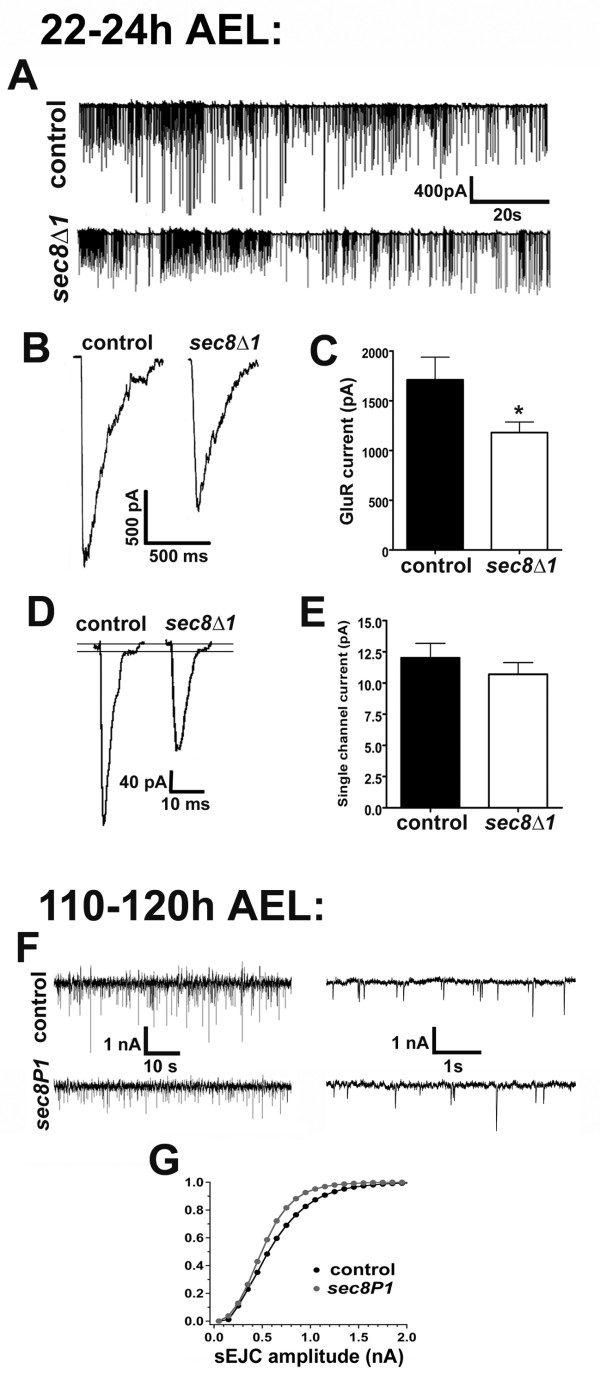
Fewer synaptic glutamate receptors are electrophysiologically detectable in *sec8 *mutants. A: Portions of representative patch clamp recordings from control and *sec8Δ1 *mutant first instar larvae (22–24 h AEL), showing spontaneous excitatory junction currents (sEJCs) from the muscle 6 NMJ. B: Glutamate-gated currents triggered by pressure ejection of 1 mM glutamate on to patch-clamped muscle 6, from control and *sec8Δ1 *mutants. Amplitudes of glutamate-gated currents are quantified in C. C: Quantification of glutamate-gated current amplitudes from control and *sec8Δ1 *mutants. D: sEJCs from control and *sec8Δ1 *mutant first instar larvae, showing delayed single channel closings observable during the falling phase of the currents. Lines have been added to the figure to indicate the baseline and single receptor current amplitude. Single-channel glutamate receptor amplitudes were measured from delayed closings such as these; quantification of these amplitudes is shown in E. E: Quantification of single glutamate receptor current amplitudes from control and *sec8Δ1 *mutants. F: Portions of representative two-electrode voltage clamp recordings from control and *sec8P1 *third instar larvae (110–120 h AEL), showing spontaneous excitatory junction currents (sEJCs) in the muscle 6 NMJ. On the right are shown portions of recordings at expanded time scale, which sacrifices an overall impression of sEJC amplitude differences but better demonstrates the resolution with which larval sEJCs were detected. G: Cumulative frequency histogram of sEJC amplitudes in third instar animals. Homozygous *sec8P1 *mutant animals had fewer large events, compared with controls.

Sec8 has been suggested to play a role in calcium-dependent neurotransmitter secretion [11]. To determine whether transmission in *sec8 *mutants is functionally abnormal, we used voltage clamp synaptic electrophysiology on NMJs. First we examined *sec8Δ1 *mutants, since Sec8 was almost completely eliminated from the NMJ in these animals (Fig. 2B). Whole-cell patch clamp recordings from homozygous *sec8Δ1 *mutants showed that *sec8Δ1 *mutant NMJs were capable of robust endogenous synaptic transmission (Fig. 4A). This result is consistent with the fact that newly hatched *sec8Δ1 *mutant larvae appeared to crawl and feed efficiently. The frequency of spontaneous synaptic currents (aka 'spontaneous excitatory junction currents', or 'sEJCs') was normal in *sec8Δ1 *mutants (Control = 13.30 ± 1.25 Hz, n = 11; *sec8Δ1 *= 12.34 ± 2.78 Hz, n = 10, p = 0.750). These data suggest no major disruption of presynaptic function after loss of Sec8. However, sEJC amplitude in *sec8Δ1 *mutant first instar NMJs was mildly but significantly reduced (K-S statistic = 0.379, p < 0.0001). Reduced sEJC amplitude suggests loss or mislocalization of postsynaptic glutamate receptors.

To test glutamate receptor function directly, we pressure ejected 1 mM glutamate on to patch-clamped (-60 mV) postsynaptic muscle. Pressure ejection of glutamate provides a means of measuring postsynaptic receptor function independent of presynaptic glutamate release. *Drosophila *NMJ receptors have low affinity for glutamate compared to mammalian glutamate receptors [34]; 1 mM is barely saturating. Currents triggered by pressure ejection of glutamate were significantly reduced in *sec8Δ1 *mutants compared to controls (Fig. 4B–C; Control = 1858 ± 222.4 pA, n = 13; *sec8Δ1 *= 1181 ± 105.5 pA, n = 11, p = 0.016). This reduction in glutamate-gated current amplitude could be due to a decrease in the number of functional postsynaptic glutamate receptors, or a change in the biophysical properties of individual receptors. In embryonic/L1 *Drosophila*, these two possibilities can be distinguished by measuring the single channel current size of synaptic receptors directly. The input resistance of embryonic/L1 *Drosophila *muscle cells, coupled with the large conductance of insect muscle glutamate receptors, allows whole-cell patch clamp mode discrimination of delayed single channel closing on the falling phase of some sEJCs [35-38]. The amplitude of these delayed synaptic receptor closings was not significantly different between *sec8Δ1 *mutants and controls (Fig. 4D–E; control = 12.02 ± 1.15 pA, n = 11; *sec8Δ1 *= 10.70 ± 0.94 pA, n = 10, p = 0.392), suggesting that the decrease in glutamate-gated current size measured in *sec8 *mutants is due to a loss of functional postsynaptic receptors, rather than a change in receptor properties. These latter experiments could not be repeated in *sec8P1 *mutant third instar larvae because synaptic receptors are not accessible for rapid agonist application because presynaptic terminals tend to be embedded deep within a subsynaptic reticulum, SSR. Decreased muscle input resistance in L3 animals also makes it impossible to discriminate single channel current amplitudes. However, two-electrode voltage clamp recordings revealed that, as in *sec8Δ1 *mutant L1 animals, sEJC amplitude was significantly reduced in *sec8P1 *mutant third instar larvae (Fig. 4F–G; K-S statistic = 0.815, p < 0.0001), consistent with loss of postsynaptic glutamate receptors in both mutant alleles throughout larval development.

To confirm the electrophysiological results and determine whether loss of receptors in *sec8 *mutants might be due to loss of a specific receptor subtype, we performed immunocytochemical experiments on the same synapses examined electrophysiologically (muscle 6 NMJs). *Drosophila *NMJs contain two spatially, pharmacologically and biophysically distinct subtypes of postsynaptic glutamate receptor [38-42], referred to as 'A-type' and 'B-type' receptors. A and B-type receptors appear to be molecularly identical heterotetramers that differ by one subunit: A-type receptors contain the subunit GluRIIA, but not GluRIIB, and B-type receptors contain the subunit GluRIIB, but not GluRIIA. Both receptor subtypes also contain the subunits GluRIIC (aka GluRIII), GluRIID and GluRIIE [39-41].

**Figure 5:**
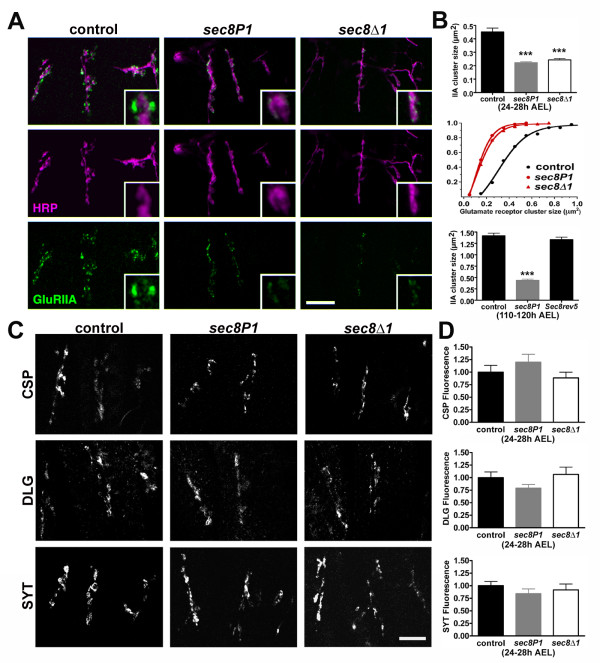
Fewer glutamate receptors are immunocytochemically detectable in *sec8 *mutants. A: Confocal images showing NMJs on ventral longitudinal muscles 15, 6/7, 13 and 12 in first instar larvae (24–28 h AEL). NMJs were visualized using the neuronal membrane marker anti-HRP (magenta). Postsynaptic glutamate receptors were visualized using antibodies raised against the glutamate receptor subunit GluRIIA (green). Scale bar: 10 μm. Quantification of GluRIIA cluster sizes is shown in B. B: Top: Average GluRIIA cluster size was significantly reduced in *sec8 *mutant first instar larvae. Middle: Frequency histogram of GluRIIA cluster sizes. Bottom: Average GluRIIA cluster size was significantly reduced in *sec8P1 *mutant third instar larvae, and this phenotype is rescued by a precise excision of the P-element ('*sec8rev5*'). Similar results were obtained using antibodies against GluRIIB and GluRIIC (see text). C: Confocal images showing ventral longitudinal NMJs in first instar larvae, as in A, stained using antibodies against cysteine string protein (CSP; top panels), discs-large (DLG; middle panels), and synaptotagmin (SYT; bottom panels). Scale bar: 10 μm. D: Quantification of staining in C. *sec8 *mutants showed no significant difference in cysteine string protein (CSP, top), discs-large (DLG, middle) or synaptotagmin (SYT, bottom) immunofluorescence compared with controls.

We visualized A-type glutamate receptors using an antibody against the receptor subunit GluRIIA. We visualized B-type receptors using an antibody against the receptor subunit GluRIIB. We also used an antibody against the subunit GluRIIC, which is shared by both glutamate receptor subtypes. At *Drosophila *embryonic/L1 NMJs, glutamate receptor subunit immunoreactivity is visible as small puncta clustered under and around presynaptic terminals [37,38,40-42]. Figure 5A shows embryonic NMJs double-stained using anti-HRP antibodies to visualize presynaptic nerve terminals (magenta) and anti-GluRIIA antibodies to visualize A-type postsynaptic glutamate receptors (green). In *sec8P1 *and *sec8Δ1 *mutant first instar larvae, the cluster size of both A- and B-type glutamate receptors was significantly reduced (Fig. 5A–B; GluRIIA Control cluster sizes = 0.45 ± 0.02 μm^2^, n = 115 clusters in 9 animals; *sec8P1 *= 0.22 ± 0.01 μm^2^, n = 124 clusters in 11 animals, p < 0.0001; *sec8Δ1 *= 0.24 ± 0.01 μm^2^, n = 142 clusters in 11 animals; GluRIIB Control cluster sizes = 0.32 ± 0.01 μm^2^, n = 117 clusters in 9 animals; *sec8P1 *= 0.24 ± 0.01 μm^2^, n = 126 clusters in 11 animals, p < 0.0001; *sec8Δ1 *= 0.25 ± 0.01 μm^2^, n = 142 clusters in 11 animals; GluRIIC control cluster sizes = 0.32 ± 0.01 μm^2^, n = 140 clusters in 9 animals; *sec8P1 *= 0.26 ± 0.01 μm^2^, n = 139 clusters in 11 animals, p < 0.0001; *sec8Δ1 *= 0.22 ± 0.01 μm^2^, n = 141 clusters in 11 animals). Glutamate receptor cluster sizes were also significantly reduced in *sec8P1 *mutant third instar larvae (Fig. 5B, bottom; Control GluRIIA cluster sizes in third instar larvae = 1.42 ± 0.06 μm^2^, n = 120 clusters in 9 animals; *sec8P1 *= 0.44 ± 0.03 μm^2^, n = 120 clusters in 9 animals; p < 0.0001; Control GluRIIB cluster sizes = 0.82 ± 0.05 μm^2^, n = 160 clusters in 11 animals; *sec8P1 *GluRIIB cluster sizes = 0.47 ± 0.02 μm^2^, n = 150 clusters in 11 animals; p < 0.0001; Control GluRIIC cluster sizes = 1.52 ± 0.06 μm^2^, n = 90 clusters in 6 animals; *sec8P1 *GluRIIC cluster sizes = 0.98 ± 0.05 μm^2^, n = 90 clusters in 6 animals; p < 0.0001). Revertant third instar larvae showed no change in glutamate receptor cluster size (Fig. 5B, bottom; Control GluRIIA cluster sizes = 1.42 ± 0.06 μm^2^, n = 120 clusters in 9 animals; sec *8rev5 *= 1.33 ± 0.06 μm^2^, n = 140 clusters in 11 animals, p = 0.303).

We also quantified the immunoreactivity of the presynaptic proteins cysteine string protein (CSP) and synaptotagmin (SYT), as well as the MAGUK protein discs-large (DLG), which is primarily localized postsynaptically. These antigens do not form distinct immunoreactive clusters. Therefore, we quantified CSP, SYT and DLG immunoreactivity by measuring average fluorescence intensity in the NMJ minus average non-NMJ muscle background intensity over an identical area of neighboring muscle membrane, normalized to control genotype (e.g.: immunoreactivity = (f_NMJ _- f_muscle_)_mutant _/ (f_NMJ_-f_muscle_)_control_). This approach avoids use of fluorescence intensity from a different wavelength as a control, which would be inappropriate because fluorescence of different fluorophores and detection of different emission spectra can vary independently from that of the 'target' fluorophore. Immunoreactivity for CSP, DLG, and SYT was normal in both *sec8P1 *and *sec8Δ1 *(Fig. 5C–D; CSP fluorescence control = 1.0 ± 0.13, n = 14; *sec8P1 *= 1.19 ± 0.15, n = 14, p = 0.351; *sec8Δ1 *= 0.88 ± 0.11, n = 14, p = 0.519; DLG fluorescence control = 1.0 ± 0.11, n = 14; *sec8P1 *= 0.79 ± 0.07, n = 14, p = 0.132; *sec8Δ1 *= 1.06 ± 0.14, n = 14, p = 0.732; SYT fluorescence control = 1.0 ± 0.08, n = 14; *sec8P1 *= 0.84 ± 0.09, n = 14, p = 0.223; *sec8Δ1 *= 0.91 ± 0.11, n = 14, p = 0.571). Since CSP and SYT are critical for normal synaptic transmission [43], and DLG is required for proper localization of GluRIIB [38,44], normal immunoreactivity for CSP, SYT and DLG is consistent with relatively normal neurotransmission and GluRIIB clustering that we observed.

Taken together, our electrophysiological and immunocytochemical results suggest that *sec8 *mutant NMJs are capable of relatively normal neurotransmission, except for a slight loss of postsynaptic glutamate receptors.

### The loss of glutamate receptors in *sec8 *mutant NMJs is due to mislocalization of glutamate receptor protein

Although the loss of postsynaptic glutamate receptors that we observed in *sec8 *mutants was slight, we felt this phenotype deserved further investigation since Sec8 has been implicated in glutamate receptor trafficking, but Sec8-dependent trafficking of non-NMDA receptors has not been demonstrated [12-14]. *Drosophila *NMJ glutamate receptors are most similar in amino acid sequence to mammalian non-NMDA kainate receptors [40,41]. The *Drosophila *genome also encodes NMDA receptor subunits, which are expressed in the CNS but not in muscle [45,46].

**Figure 6:**
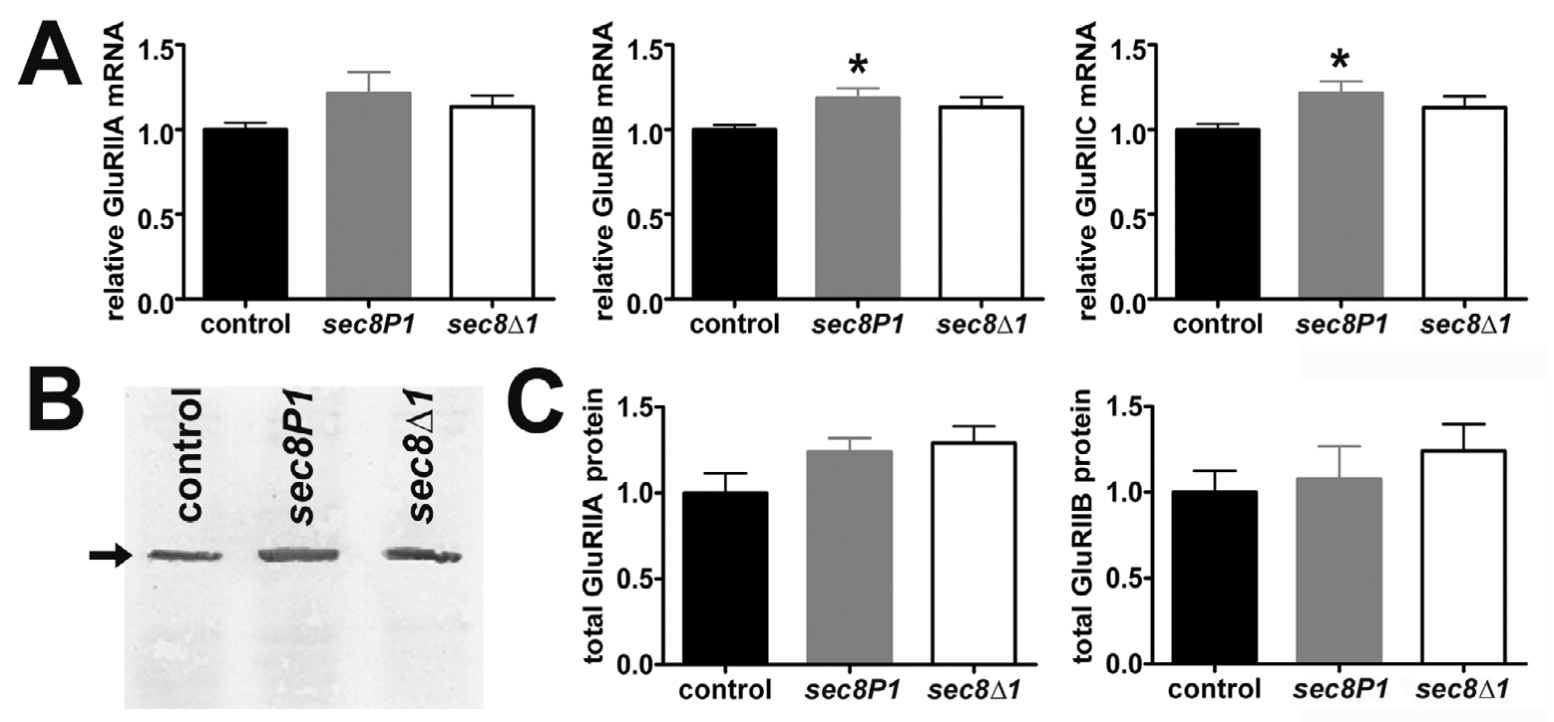
Glutamate receptor subunit mRNA and protein is not reduced in *sec8 *mutants. A: Relative mRNA levels for glutamate receptor subunits GluRIIA (left), GluRIIB (middle), and GluRIIC (right), as measured by quantitative real-time RT-PCR in control and *sec8 *mutant first instar larvae. B: Example immunoblot from first instar larvae (24–28 h AEL) probed with a anti-GluRIIB antibody. The arrow points to the GluRIIB band. GluRIIA immunoblots have been previously described [71]. GluRIIA and GluRIIB bands are eliminated in Df(2L)SP22 mutants, in which GluRIIA and GluRIIB genes are deleted (not shown). Loading control was total protein; to ensure that the same amount of protein was loaded for both control and mutant, total extracted protein quantity was measured using a Bradford assay and then the same amount of protein was loaded on to the gel for each mutant (either 20 μg for GluRIIB blots or 50 μg for GluRIIA blots). Loading was also checked with Coomassie staining (not shown). C: Quantification of total GluRIIA (left) and GluRIIB (right) protein, as measured from immunoblots such as the one shown in B.

To determine whether the loss of postsynaptic receptors might be due to changes in receptor subunit expression, we measured glutamate receptor subunit mRNA levels using quantitative real-time RT-PCR. In embryonic/L1 *sec8 *mutants, the relative levels of GluRIIA, GluRIIB and GluRIIC mRNA were not reduced compared to controls (Fig. 6A; GluRIIA: Control = 1.0 ± 0.03 arbitrary units (a.u.), n = 7; *sec8P1 *= 1.21 ± 0.12 a.u., n = 8, p = 0.153; *sec8Δ1 *= 1.13 ± 0.06 a.u., n = 10, p = 0.128; GluRIIB: Control = 1.0 ± 0.02 a.u., n = 8; *sec8P1 *= 1.18 ± 0.05 a.u., n = 9, p = 0.016; *sec8Δ1 *= 1.13 ± 0.05 a.u., n = 10, p = 0.067; GluRIIC: Control = 1.0 ± 0.03 a.u., n = 8; *sec8P1 *= 1.21 ± 0.07 a.u., n = 9, p = 0.018; *sec8Δ1 *= 1.13 ± 0.06 a.u., n = 10, p = 0.129).

To determine whether the loss of postsynaptic receptors involved decreased glutamate receptor protein production or increased degradation, we measured total GluRIIA and GluRIIB subunit protein using immunoblots. GluRIIA and GluRIIB are thought to be expressed only in body wall muscle [40,47,48]. Immunoblot analysis suggested a reduction in neither total GluRIIA nor total GluRIIB protein in *sec8 *mutants (Fig. 6B–C; GluRIIA Control = 1.0 ± 0.11 a.u., n = 6; *sec8P1 *= 1.24 ± 0.07 a.u., n = 6, p = 0.114; *sec8Δ1 *= 1.29 ± 0.09 a.u., n = 6, p = 0.085; GluRIIB Control = 1.0 ± 0.12 a.u., n = 5; *sec8P1 *= 1.07 ± 0.19 a.u., n = 6, p = 0.754; *sec8Δ1 *= 1.24 ± 0.15 a.u., n = 5, p = 0.254). This result suggests that, in *sec8 *mutants, glutamate receptor protein may be dispersed throughout the cell instead of appropriately localized in the postsynaptic membrane, where it would have been detectable immunocytochemically and electrophysiologically. These results, in combination with our electrophysiology and immunocytochemistry, are consistent with a minor role for Sec8 in non-NMDA glutamate receptor trafficking.

### *Drosophila *Sec8 inhibits microtubule formation *in vivo*

**Figure 7:**
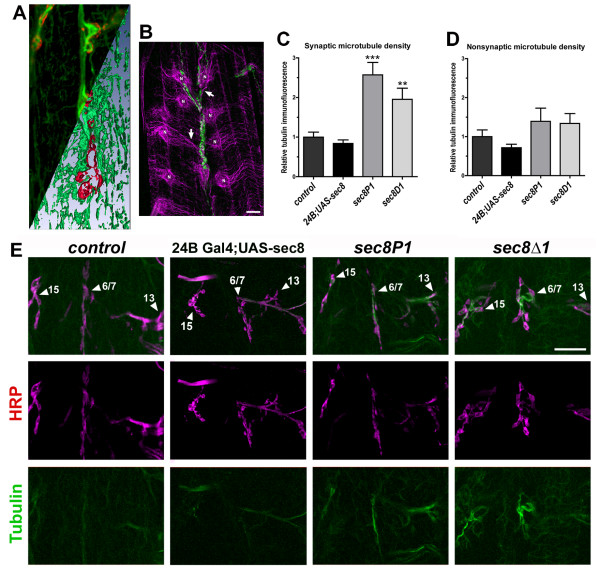
Microtubule density is inversely proportional to Sec8 expression. A: Confocal images showing a portion of a NMJ on ventral longitudinal muscles 6 & 7 in third instar larvae (110–120 h AEL), visualized using antibodies against tubulin (green) and the essential glutamate receptor subunit GluRIIC (red). Upper left half of the image shows confocal projection; lower right half of the image shows 3D isosurface reconstruction of the same data. This shows the close association between pre and postsynaptic microtubules and the NMJ. B: Microtubules in postsynaptic larval muscles. Muscle nuclei ('N') are surrounded by microtubule networks (labeled with anti-acetylated tubulin antibody, magenta) that often extend (arrows) toward the neuromuscular junction (labeled with anti-HRP, green). This suggests that muscle microtubules may be critical for delivery of postsynaptic molecules to the NMJ. C: Quantification of synaptic microtubule density. To measure synaptic microtubule density, immunofluorescence from only the non-axonal area delimited by HRP staining (magenta) was measured. D: Quantification of extrasynaptic microtubule density. To measure extrasynaptic microtubule density, immunofluorescence from the muscle area not delimited by HRP staining was measured. E: Confocal images showing intersegmental nerve branch b (ISNb) innervating ventral longitudinal muscles in first instar larvae (24–28 h AEL). Neuronal tissue was visualized using anti-HRP antibody (magenta). Microtubules were visualized using an anti-acetylated tubulin antibody, which recognizes only stably polymerized alpha and beta tubulin (green). Individual NMJs are identified (arrows, labels). In *24B Gal4;UAS-sec8 *animals, *sec8 *cDNA is overexpressed in postsynaptic muscle cells, resulting in small but not statistically significant reductions in microtubule immunofluorescence. In *sec8 *mutants, microtubule immunofluorescence is increased. This increase is highest at the synapse, where Sec8 is normally localized. Qualitatively identical results were obtained using another antibody that recognizes all tubulin (not shown). Scale bar: 10 μm.

Previous work has demonstrated that exocyst proteins associate biochemically with microtubule proteins [10], and that this association destabilizes microtubules [15]. To test whether Sec8 regulates microtubules at the *Drosophila *NMJ, we examined pre and postsynaptic NMJ microtubules using an antibody against acetylated tubulin, which preferentially recognizes polymerized tubulin. Microtubules are abundant in presynaptic motor axon terminals and throughout postsynaptic muscle cells (Fig. 7A–B and [49-52]). Both pre- and postsynaptically, microtubules are intimately associated with the NMJ (Fig. 7A–B and [49-52]). Within the *Drosophila *presynaptic motor terminal, microtubules form distinct loops that appear to be critical for presynaptic arborization and regulation of bouton growth [49,50,53,54]. Postsynaptic muscle microtubules also regulate NMJ growth and development [51,52]. In L1 animals overexpressing *sec8 *cDNA in muscles, microtubule immunoreactivity was noticeably decreased (Fig. 7E), but this decrease was not statistically significant (Fig. 7C–D; Control MT density = 1.00 ± 0.17, N = 11; *24B Gal4;UAS sec-8 (*muscle overexpression of *sec8*) = 0.72 ± 0.09, N = 13, p = 0.14).

In contrast, microtubule immunofluorescence at NMJs was approximately doubled in *sec8P1 *and *sec8Δ1 *L1 mutant larvae (Fig. 7C,E; Control synaptic MT density = 1.00 ± 0.12, N = 11; *sec8P1 *= 2.57 ± 0.31, N = 11, p = 0.0002; *sec8Δ1 *= 1.95 ± 0.28, N = 11, p = 0.005). Very little Sec8 is localized extrasynaptically in wildtype animals. Therefore, a change in synaptic microtubule immunofluorescence (where Sec8 is normally found), but not in extrasynaptic microtubule immunofluorescence (where only very little Sec8 is found), is most consistent with synapse-specific regulation of microtubules by Sec8. Extrasynaptic microtubule immunofluorescence was not significantly increased in either *sec8P1 *or *sec8Δ1 *L1 mutant larvae (Fig. 7D,E; Control extrasynaptic MT density = 1.00 ± 0.17, N = 11; *sec8P1*= 1.39 ± 0.33, N = 11, p = 0.31; *sec8Δ1 *= 1.33 ± 0.25, N = 11, p = 0.28). We conclude from these data that Sec8 inhibits microtubules in vivo, as predicted by previous biochemical data showing inhibition of microtubule formation by sec proteins [10,15].

### Overexpression of tubulin phenocopies the loss of glutamate receptors seen in *sec8 *mutants

**Figure 8:**
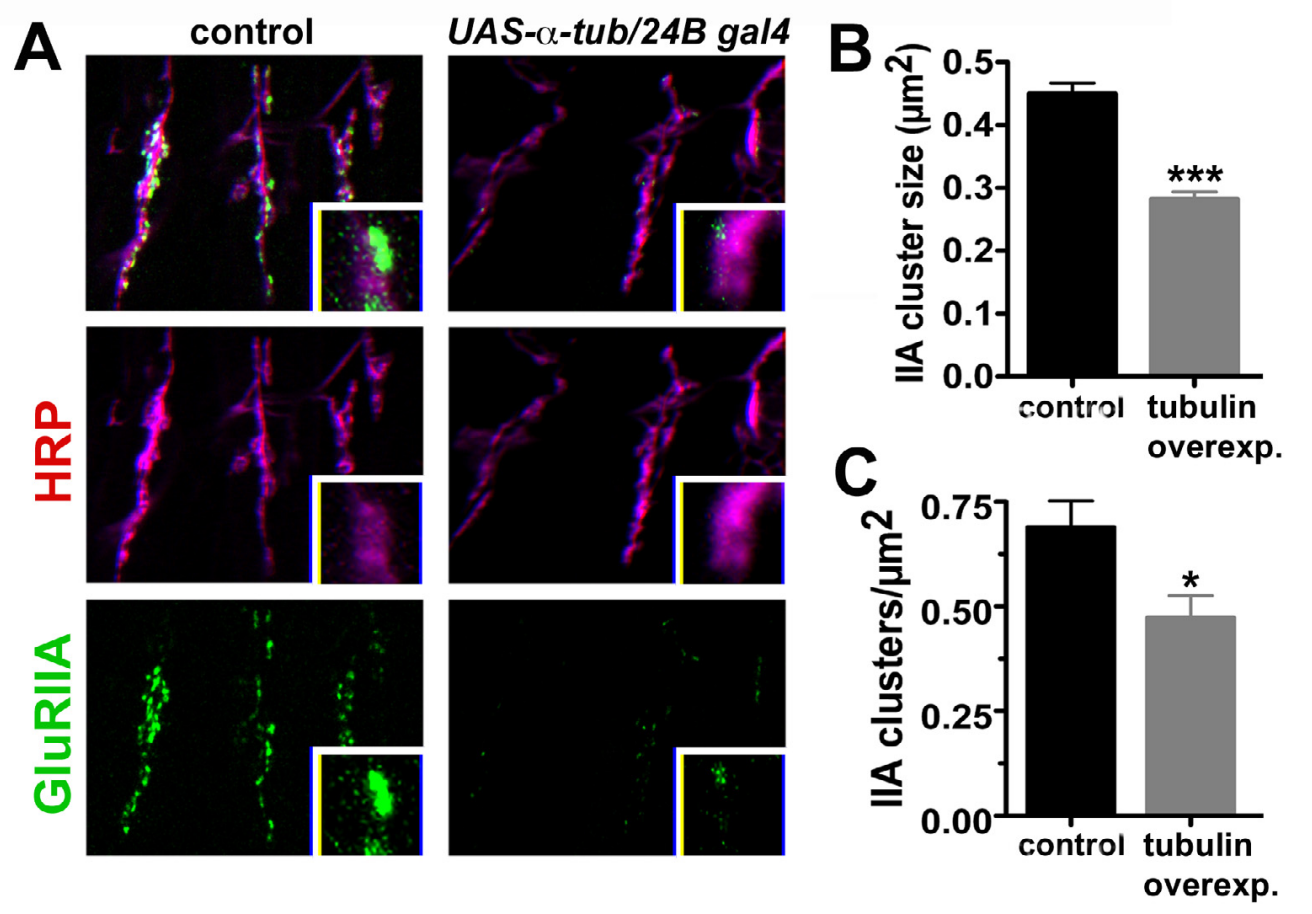
Glutamate receptor clustering is inhibited by overexpression of tubulin. Overexpression of alpha tubulin in muscles triggers an increase in microtubules (see text). A: Confocal images showing intersegmental nerve branch b (ISNb) innervating ventral longitudinal muscles in first instar larvae (24–28 h AEL). Neuronal tissue was visualized using anti-HRP antibody (magenta). Glutamate receptors were visualized using an antibody against the receptor subunit GluRIIA (green). In *UAS-alpha tubulin/24B gal4 *animals, *sec8 *cDNA is overexpressed in postsynaptic muscle cells. Overexpression of tubulin causes a reduction in glutamate receptor cluster size and number (quantified in B and C). B: Quantification of receptor cluster size, showing a reduction in cluster size after postsynaptic overexpression of tubulin (*UAS-alpha tubulin/24B gal4*). C: Quantification of cluster number, showing a reduction in cluster number after postsynaptic overexpression of tubulin (*UAS-alpha tubulin/24B gal4*).

Our data suggest that *Drosophila *Sec8 plays a role in glutamate receptor localization and microtubule regulation. These two phenotypes could represent separate functions for Sec8, or could be due to the same root cause. Mammalian glutamate receptors are trafficked to the synapse via the microtubule network [55-57]. Therefore, it is reasonable to surmise that the defects in postsynaptic glutamate receptor localization measured in *sec8 *mutants might be due to misregulation of microtubules. To address this, we mimicked the increase in microtubules observed in *sec8 *mutants by overexpressing alpha tubulin. Overexpression of an alpha tubulin transgene in postsynaptic muscles using the gal4-UAS system doubles microtubule density (Images not shown; Control MT density = 1.00 ± 0.17, N = 11; *UAS-α tub/B24 gal4 *tubulin = 2.21 ± 0.24 a.u., n = 12, p = 0.001), presumably due to a shift in the dynamic equilibrium between disassembled and assembled tubulin subunits. This shift toward an increase in microtubules caused a significant decrease in the size and number of postsynaptic glutamate receptor clusters, similar to that observed in *sec8 *mutants (Fig. 8; Control GluRIIA cluster size = 0.45 ± 0.02 μm^2^, n = 115 clusters in 8 animals; *UAS-α tub/B24 gal4 *= 0.28 ± 0.01 μm^2^, n = 104 clusters in 9 animals, p < 0.0001; Number of control clusters = 0.68 ± 0.06 clusters/μm^2^, n = 13 NMJs; *UAS-α tub/B24 gal4 *= 0.47 ± 0.05 clusters/μm^2^, n = 14 NMJs, p = 0.014). These results suggest that the loss of postsynaptic glutamate receptors observed in *sec8 *mutants may be secondary to disruption of microtubule networks.

## Discussion

Ours is the first detailed examination of any multicellular Sec8 mutant. Sec proteins are thought to play a role in almost every cell type, but are particularly highly enriched in the brain. Therefore, most interest in Sec protein function is within the context of neuronal development and function. Sec8, alone or as part of the exocyst complex, has been implicated in several aspects of neural development and function including: (1) neurite outgrowth [7-10], (2) calcium-dependent neurotransmitter secretion [11], (3) trafficking of ionotropic glutamate receptors [12-14] and (4) regulation of neuronal microtubule assembly [10,15]. We explored each of these areas of potential function in *Drosophila sec8 *mutants.

Surprisingly, we did not see any evidence that Sec8 is required for neurite extension or nerve terminal growth, at least in motor neurons. Instead, *sec8 *mutant NMJ motor arborizations were morphologically normal at time of hatching, but then developed more branches and more boutons during larval development, compared to controls. This suggests that Sec8 *suppresses *neurite growth. The *sec8 *mutant NMJ overgrowth phenotype is unlikely to be an artifact for several reasons: (1) Although our immunoblots showed that 50% of total late embryonic/L1 Sec8 was left after deletion of the gene, Sec8 in the NMJ at the start of larval development was already almost undetectable. (2) Regardless of whether the amount of Sec8 is reduced to 50% or 0% (the difference between a functional hypomorph or a null), there was no detectable impairment of presynaptic terminal growth. Instead, NMJ growth was enhanced in the sec8 mutants. (3) The NMJ growth enhancement occurred entirely during larval development – when maternal sec8 was fading to its lowest levels. This *sec8 *mutant NMJ overgrowth phenotype is dramatically different from the phenotype observed in *sec5 *mutants [7]. *Drosophila sec5 *mutants showed a slight increase in NMJ growth during L1 development (c.f. Murthy et al., Fig 3B), but then progressive deterioration in the ability to form new motor nerve terminals through later larval development [7]. This suggests, along with other evidence (see below), that metazoan Sec proteins may not always function together as part of a canonical octomeric complex.

Because Sec proteins are required for membrane secretion in yeast, neuronal Sec proteins have long been implicated in neurotransmission, which relies on synaptic vesicle membrane insertion and cycling. However, we did not see any evidence that Sec8 is critical for neurotransmitter secretion, consistent with recently published descriptions of *Drosophila sec5 *and *sec15 *mutants [7,21], and *sec10 *RNAi expression in the fly NMJ [58]. Our result is unlikely to be an artifact of incomplete removal of Sec8 from the synapse, because even though *sec8 *zygotic null mutants showed substantial maternal contribution of Sec8 protein (~ 50% normal levels overall), immunocytochemistry and confocal microscopy showed that Sec8 was almost completely absent from mutant early larval NMJs. Thus, we were able to examine synaptic function following severe genetic reductions in synaptic Sec8. No mutant evidence to date suggests a role for Sec proteins in neurotransmission. Our data add Sec8 to the list of tested Sec proteins, and strengthen this conclusion.

Mammalian Sec8 has been shown to interact with NMDA-type glutamate receptors and the PDZ-domain protein SAP102 [12]. This interaction appears critical for delivery of NMDA receptors to synaptic membrane in cultured cells [12]. Sec8 also interacts with PSD-95 and SAP-97 [13,14], which are two proteins thought to be important for delivery of non-NMDA (AMPA & kainate) glutamate receptors to postsynaptic membranes. Thus, Sec8 has been implicated in trafficking of all types of glutamate receptors. We observed mislocalization of postsynaptic glutamate receptors in *Drosophila sec8 *mutants, consistent with a role for Sec8 in glutamate receptor trafficking. However, the mechanism by which *Drosophila *Sec8 regulates glutamate receptor trafficking may not be the same as the mechanism proposed for mammalian neurons. As mentioned above, mammalian glutamate receptor trafficking by Sec8 is thought to occur via interactions with the PDZ-domain proteins SAP102, PSD-95 or SAP-97. DLG is the sole *Drosophila *member of the mammalian PSD-95/SAP97/SAP102 protein family [59-61]. In *Drosophila *DLG mutants, only B-type receptor trafficking/localization is disrupted; trafficking and localization of A-type receptors appear normal [38]. But in the *sec8 *mutants described here, trafficking of both A- and B-type receptors was affected. Therefore, our evidence suggests that loss of Sec8 does not selectively disrupt a DLG-dependent glutamate receptor trafficking pathway, which would be expected to lead to loss of only B-type receptors.

Exocyst proteins, including Sec8, have recently been shown to associate with microtubules in vitro and in cultured cells, and overexpression of sec proteins inhibits microtubule polymerization [10,15]. Overexpression of *Drosophila sec8 *cDNA in vivo also caused a noticeable but statistically insignificant reduction in microtubule immunoreactivity, and *sec8 *loss of function mutants showed dramatically increased microtubule immunoreactivity in NMJs. These results suggest that Sec8, probably in conjunction with other sec proteins, locally inhibits synaptic microtubule network assembly and/or stability.

The misregulation of microtubules in *sec8 *mutants could be responsible for the glutamate receptor mislocalization we observed, since glutamate receptors, like other transmembrane proteins, are thought to be transported to synapses via microtubule networks [55-57]. Consistent with the idea that the receptor trafficking defects are secondary to microtubule misregulation, postsynaptic overexpression of tubulin increased muscle microtubule density and phenocopied the loss of synaptic glutamate receptors measured in *sec8Δ 1 *mutants. Misregulation of microtubules in sec8mutants might also explain the presynaptic growth defects that we observed in *sec8 *mutants. Presynaptic growth in the *Drosophila *NMJ is known to be dependent on microtubule stability in both pre and postsynaptic cells [49-54]. Misregulation of microtubules by either pre or postsynaptic Sec8 might therefore be expected to trigger defects in presynaptic growth.

Regulation of microtubules by Sec8 might also explain why expression of a *sec8 *transgene in *sec8 *mutant neurons (*elav C155 gal4/UAS-sec8; sec8*) fails to rescue the synaptic overgrowth (Data not shown), and why overexpression of a *sec8 *transgene in *sec8 *mutant muscle (*UAS-sec8; 24B gal4 *or *UAS-sec8; H94 gal4*) fails to reverse *sec8 *mutant receptor trafficking phenotypes (Data not shown). Traditionally, these results might be taken as genetic evidence that our phenotypes may be due to 'background' mutations. But nervous-system-specific expression *sec8 *(*elav C155 gal4/UAS-sec8; sec8*) rescues the mutant viability, *sec8P1 *mutants retain their phenotype in trans to a deficiency (*Df(3R)Tpl10*) that removes the *sec8 *gene region, and precise excision of the P-element in *sec8P1 *mutants completely reverses all synaptic phenotypes (see results). These results all argue that: (1) The *sec8 *mutant flies die owing to problems in the nervous system but not the NMJ defects, and (2) all NMJ phenotypes are indeed due to specific disruption of *sec8*.

The failure of transgenic rescue is also consistent with the fact that overexpression of *sec8 *(in a wildtype background) phenocopies *sec8 *mutants. For example, presynaptic overexpression of *sec8 *causes NMJ overgrowth (Data not shown; Control boutons per 6/7 NMJ = 38.45 ± 1.65, n = 22; *elav C155 gal4/UAS-sec8 *= 37.11 ± 2.51, n = 18, p = 0.647; Control branches per 6/7 NMJ = 3.13 ± 0.30, n = 22; *elav C155 gal4/UAS-sec8 *= 4.61 ± 0.36 n = 18, p = 0.003). Interestingly, postsynaptic overexpression of sec8 also causes NMJ overgrowth (Data not shown; Control boutons per 6/7 NMJ = 38.45 ± 1.65, n = 22; *UAS-sec8; 24B gal4 *= 53.80 ± 3.19, n = 20, p < 0.0001; control branches per 6/7 NMJ = 3.13 ± 0.30, n = 22; *UAS-sec8; 24B gal4 *= 7.15 ± 0.51, n = 20, p < 0.0001). This latter result is consistent with the idea that Sec8 regulates microtubules and the knowledge that microtubule stability – on both the pre and postsynaptic sides of the NMJ- is a critical regulator of presynaptic terminal growth and arborization [49-54].

Considered together, our results suggest that a primary role for Sec8 at synapses may be spatially-restricted regulation of microtubule networks. But we cannot conclude that this is a primary function for other Sec proteins. A growing body of evidence suggests that metazoan exocyst proteins may not always function as a canonical hetero-octomeric complex; functionally distinct subcomplexes appear to exist in different cell types or even within the same cell [21,23,62-64]. There are, for example, important differences between the *sec8 *mutant phenotypes described here and the phenotypes previously described for *Drosophila *Sec5 mutants with regard to NMJ formation [7,22]. Similarly, *Drosophila *expressing *sec10 *RNAi in the neuromusculature showed no NMJ changes, and Sec10 was not detected in the NMJ [58]. More recently, a study of *Drosophila *Sec15 mutants found more differences between *sec *mutant phenotypes and argued explicitly for independent Sec protein roles [21]. Thus, several lines of evidence, including expression patterns and phenotypes, suggest that Sec proteins function independently in at least some tissues and/or at different times. Nevertheless, all *Drosophila sec *mutants show at least partial defects in membrane trafficking, membrane protein distribution and/or cell polarization – processes that rely, at least to some extent, on microtubules. Thus, microtubule regulation may be a common thread tying together Sec protein function in vivo. One might imagine that different subsets of differentially-regulated Sec proteins transiently associate in specific cellular compartments to prune the dynamic microtubule network. This pruning and shaping might ensure that membrane packages are delivered past, or are dropped off at, appropriate places. We think that it is important for future work on Sec proteins to consider this possibility.

## Conclusion

Sec8 is abundant in the *Drosophila *NMJ. Sec8 is required in vivo for regulation of synaptic microtubule formation, and (probably secondarily) regulation of synaptic growth and glutamate receptor trafficking. We did not find any evidence that Sec8 is required for basal neurotransmission.

## Methods

### Genetics

To identify genes required for glutamatergic synapse development, we screened *GT1 *and *SuPor-P *transposon insertions from the Berkeley *Drosophila *Gene Disruption Project [17]. Specifically, we first identified homozygous mutants using the FlyBase Insertions Query Form . Approximately 10% (220 of 2185 stocks) of the insertion lines available in March 2003 contained lethal inserts. We examined dechorionated embryos from each of the 220 stocks to ensure that homozygous mutants developed to at least late stage embryogenesis (16–17 h after egg laying, AEL), which is when NMJs begin to form. Of the 220 mutants, 202 developed into morphologically mature embryos (condensed CNS, clear segmentation, trachea, mouth hooks, etc.). These 202 mutants were rebalanced using a chromosome-specific GFP balancer for unambiguous identification of homozygous *P*-element mutants and then screened immunocytochemically using anti-HRP and anti-GluRIIA antibodies to visualize presynaptic nerve terminals and postsynaptic glutamate receptors, respectively. The remaining 18 stocks were discarded from future consideration. *P{SUPor-P}CG2095*^*KG02723 *^mutants (herein referred to as '*sec8P1*') were identified as a member of the subgroup of mutants with defects in both presynaptic morphology and postsynaptic glutamate receptor expression. A more complete description of this screen, and the mutants identified therein, can be found elsewhere [5,6].

*sec8Δ1 *mutants were created by imprecise excision of *P{SUPor-P}CG2095*^*KG02723 *^from *sec8P1*, using standard methods for *P*-element mobilization [18]. Four hundred and forty-five independent mutant lines of flies were created; approximately 8% of these (35 of 445) were homozygous lethal. The lethal lines were then screened using a PCR-based assay with three pairs of primers. One pair of primers was designed to amplify a portion of *sec8 *near the *P*-element insertion. A second set of primers was designed to amplify a portion of the gene *CG2082*, located immediately 5' to *CG2095*, and the third set of primers was designed to amplify a portion of gene *CG2091*, located immediately 3' to *sec8*. Mutants in which the first (*sec8 *specific) primer pair did not produce a product but the second and third primer sets did suggested that *sec8 *was specifically deleted but the neighboring genes were left intact. Eight mutants of this type were identified. Lethal lines containing partial deletions of *sec8 *were further characterized using several sets of PCR primers. Both forward and reverse primers were designed to every 1 kb of genomic DNA beginning 500 bp upstream and downstream of *sec8*. Using these primers, one deletion mutant (*sec8Δ1*) was isolated in which the first forward primer (positioned approximately 500 bp 5' of *sec8*) and the last reverse primer (positioned approximately 500 bp 3' of *sec8*) produced a product of approximately 1 kb. This PCR product was sequenced to determine the exact location and size of this deletion (*sec8Δ1*).

Precise excisions of *P{SUPor-P}CG2095*^*KG02723 *^('revertants') were produced by the same *P*-element mobilization that produced *sec8Δ1*. Revertants were initially identified based on eye color and reversion of the lethal insertion phenotype, then confirmed by PCR. Measurements from revertants, in which the *sec8 P*-element was precisely excised, are indicated in the text and figures. For all experiments, homozygous mutant larvae were identified by use of an appropriate GFP-tagged balancer chromosome. 'Control' genotypes in all experiments are *w*^1118^. Animals were age-matched for all experiments using timed laying plates and morphological staging of late-stage embryos.

### Molecular biology

Wild-type *CG2095 *(*sec8*) cDNA was cloned by RT-PCR amplification of embryonic RNA. Total RNA was isolated using Trizol extraction and reverse transcribed using cDNA-specific primers and cloned. The 3256 bp *sec8 *cDNA was ligated into the pUAST vector using the KpnI and XbaI sites, and then sequenced for confirmation. Our results are consistent with the predicted gene structure shown in FlyBase, although we noted several polymorphisms. We used our cDNA clone sequence to predict the CG2095 amino acid sequence. Our predicted Oregon R CG2095 amino acid sequence contains three differences compared to the predicted *CG2095 *translation product as annotated in FlyBase: Oregon R *CG2095 *encodes an M at amino acid 119, a D at amino acid 328, and a Y at amino acid 669.

Fly transformation was performed by DNA microinjection of embryos using standard methods (Genetic Services, Inc, Cambridge MA). Ten independent *UAS-sec8 *transgenic strains were created.

Quantitative real-time RT-PCR of *Drosophila *glutamate receptor subunits was performed as previously described [41]. Briefly, total RNA was isolated from 22–24 h AEL embryos using Trizol extraction and reverse transcribed using sequence specific primers. Real-time RT-PCR was then performed using subunit cDNA-specific primers in an Opticon 2 real-time cycler (MJ Research, Waltham MA), using SYBR green (Molecular Probes, Eugene, OR) for fluorescent measurement of amplicon quantity. Nonspecific fluorescence due to primers was eliminated by measuring fluorescence after a short 'holding step' at a temperature sufficient to melt primer dimers but not desired product (MJ Research Technical Note # 004). As a loading control, actin 5C RNA levels were also measured for every extraction using actin 5C-specific primers, and GluRII RNA levels normalized using this measurement. Each measurement represents the actin 5C c(t) divided by a GluRII c(t), where both measurements were made from the same RNA isolation and RT reaction.

### Immunocytochemistry

To generate Sec8 antibodies, rabbit polyclonal antisera was raised against a synthesized peptide composed of Sec8 amino acids 440–460 (GTSNNSDAFKEHRRNASDASV). The antiserum was affinity purified and used at 1:500. For staining and microscopy, animals were manually dissected and fixed for 30–60 min in either Bouin's fixative (when Sec8 or GluRII antibodies were used), or 4% paraformaldehyde (for all other staining). Note that the Sec8 antibody recently described in [21] as not showing immunoreactivity at the fly NMJ, actually does so (and shows staining very similar to our antibody) when preparations are fixed in Bouin's fixative. Mouse monoclonal anti-GluRIIA ('8B4D2', Iowa Developmental Studies Hybridoma Bank, Iowa City, IA) was used at 1:100. Rabbit polyclonal anti-GluRIIB and anti-GluRIIC [39] were gifts from Dr. Aaron DiAntonio (Washington University, St. Louis, MO) and used at 1:2000 and 1:3000, respectively. Fluorescently conjugated anti-HRP (Jackson Immunoresearch Labs, West Grove, PA) was used at 1:100. Mouse monoclonal anti-CSP was used at 1:200 [65]. Mouse monoclonal anti-Discs Large (DLG) ('4F3', Iowa Developmental Studies Hybridoma Bank, Iowa City, IA) was used at 1:1000. Mouse monoclonal anti-synaptotagmin (SYT) was used at 1:500 [66]. Mouse monoclonal anti-acetylated tubulin (Sigma) was used at 1:1000. Goat anti-rabbit or goat anti-mouse fluorescent (FITC or TRITC) secondary antibodies (Jackson Immunoresearch Labs, West Grove, PA) were used at 1:400. Confocal images were obtained using an Olympus FV500 laser-scanning confocal microscope. All images represent maximum intensity projections of confocal Z-stacks. Image analysis and quantification was performed on maximum intensity projections of Z-stacks using ImageJ software. Three-dimensional surface reconstructions were generated from confocal Z-stacks using Amira 3.1 (Mercury Computer Systems, Chelmsford, MA).

Quantification of glutamate receptor cluster size was performed as previously described [37,38,42,67]. Every cluster on a 6/7 NMJ from each animal was measured. *Drosophila *NMJ glutamate receptor clusters form over the course of hours and then stabilize to form distinct clusters with relatively invariant diameter [37,67]. These diameters stay relatively constant, even throughout larval development [30]. Because changes in the number of postsynaptic receptors within each cluster are linearly proportional to changes in cluster area; cluster area is a quantitative and sensitive optical measure of receptor number in the *Drosophila *NMJ [30,37,67]. Cluster area was quantified and measured from maximum intensity Z-projections of confocal image stacks, using automated edge finding and area measurement in ImageJ software (NIH, Bethesda MD). This method agrees well with manual measurements [37], is highly reproducible [38,42,67], and involves no experimenter bias for cluster selection because the software selects and measures every bright spot regardless of shape or size. An alternative measurement of glutamate receptor abundance is fluorescence intensity. Although fluorescence intensity measurements gave qualitatively identical results for this study (data not shown), we do not prefer them for embryonic/L1 receptor abundance measurements because intensity measurements assume changes in protein density within individual PSDs (for which we have no evidence) or underestimate differences in receptor abundance due to fluorescence measurement from pixels between individual GluR clusters. Because immunoreactivity for microtubules and the synaptic proteins DLG, CSP and SYT did not show distinct clusters, we quantified their abundance using fluorescence intensity. However, all of these measurements are relative to 'background' fluorescence (measured from non-muscle areas for microtubules, or nonsynaptic areas for DLG, CSP and SYT) in the same image and filter channel [68]. We feel that this is an important wavelength-matched control for variation due to possible differences in staining, mounting of the preparation, or microscope settings.

### Immunoblots

After protein isolation for immunoblotting, protein abundance was quantified using Bradford assays, and the same amount of protein was loaded per lane on 6% SDS-polyacrylamide gels. The amount loaded was 20 μg for GluRIIB blots, 50 μg for GluRIIA blots and 20 μg for Sec8 blots. After electrophoresis, protein was transferred to nitrocellulose membranes. Nitrocellulose membranes were blocked with 5% milk in TBST (10 mM Tris, pH 8.0, 150 mM NaCl and 0.1% Tween 20) for 1–2 h at room temperature. Anti-Sec8 was used at 1:500 and anti-GluRIIA ('8B4D2' concentrate, Iowa Developmental Studies Hybridoma Bank, Iowa City, IA) was used at 1:200. GluRIIB antibodies usable for immunoblots have not previously been described. To generate Anti-GluRIIB antibodies for use in immunoblots, rabbit polyclonal antisera was raised against synthetic peptides containing C-terminal sequence of GluRIIB: RQSRDSTSTGYSSLEQITSASSAKKKK. For immunoblotting, GluRIIB antibodies were used at 1:400. Note that these GluRIIB antibodies are not the same as used for in situ staining; as described above, the GluRIIB antibodies used for in situ staining are described in Marrus et al. [39]. After incubating at 4°C overnight, the membranes were washed with TBS-T. Anti-mouse HRP-conjugated secondary antibodies were used at 1:5000 for GluRIIA blots and anti-rabbit AP-conjugated secondaries were used at 1:1000 for GluRIIB and Sec8 blots. Protein bands on blots were visualized using enhanced chemiluminescence (GluRIIA) or via a colorimetric reaction (GluRIIB and Sec8). Both GluRIIA and GluRIIB bands are completely eliminated in appropriate mutants (Df(2L) [AD9] and Df(2L) [SP22]) [40] and Liebl, Ng, Sheng, Karr and Featherstone, unpublished).

### Electrophysiology

All electrophysiology was performed on the ventral body wall muscle 6 at 19°C. For electrophysiology on first instar (L1) larvae we used a whole-cell patch clamp technique. Third instar larval muscles are too large for whole cell patch clamp. Therefore, we used two-electrode voltage clamp technique for third instar (L3) larval recordings. Both techniques were performed as previously described [36,38,68]. The sEJCs analyzed in this study, in either first or third instar larvae, are unlikely to include events resulting from endogenous action potentials, based on two pieces of evidence: (1) Endogenous action potential evoked activity is much larger, typically patterned, and thus easily recognizable, and (2) *Drosophila *sEJCs recorded in 0 mM calcium with 5 μm TTX occur with less frequency but are not larger, in both embryos/first instars and third instar larvae [[68] and unpublished observations]. Therefore, although we do not methodologically exclude any particular type of synaptic event and therefore properly use the conservative term 'sEJC' to describe what we are measuring, all of the events shown and analyzed in this study are likely to represent only true spontaneous monovesicular 'mEJCs', or 'minis'.

For patch clamp electrophysiology on L1 (22–24 h AEL) larvae, animals were manually dissected by gluing (Histoacryl Blue, Braun Germany) them to sylgard-coated coverslips under standard *Drosophila *saline (135 mM NaCl, 5 mM KCl, 4 mM MgCl_2_, 1.8 mM CaCl_2_, 5 mM TES, 72 mM sucrose). A slit was then made along the dorsal midline using a glass capillary pulled to a sharp point, and the body walls glued flat to the coverslip. After dissection, the exposed muscle sheath was enzymatically removed by 30–90s exposure to 1 mg/ml collagenase (type IV, Sigma). Muscle 6 was then whole-cell patch clamped (-60 mV) in standard *Drosophila *saline (135 mM NaCl, 5 mM KCl, 4 mM MgCl, 1.8 mM CaCl, 5 mM TES, 72 mM sucrose) using standard patch-clamp techniques. Pressure ejection of 1 mM glutamate dissolved in extracellular saline utilized a Picospritzer III (General Valve/Parker Hannifin, Fairfield NJ). Pressure ejection pipettes were approximately the same size and tip diameter as unpolished patch pipettes. Glutamate leak was minimized by continuous back-pressure; no evidence for glutamate leak and subsequent desensitization of receptors was ever observed, and would be identical between genotypes. Data were acquired using an Axopatch 1 D amplifier and a PC running pClamp9 software (Axon Instruments, Union City, CA). Data were subsequently analyzed using Clampfit9 (Axon Instruments).

For two-electrode electrophysiology on third instar (L3) larvae (110–120 hr AEL), Dissections were made as with first instar larvae, except that collagenase was not used. Larval dissections and recordings were also performed under standard *Drosophila *saline, as described above. Standard two-electrode voltage clamp techniques were used (holding potential -60 mV), as previously described [36]. Electrodes for TEVC were filled with 3 M KCl. Data was acquired using an Axon GeneClamp 500B and a PC running Axoscope software. Data were subsequently analyzed using Clampfit9 (Axon Instruments)

In all cases (data from both L1 and L3 animals) sEJCs were detected and analyzed using Clampfit9's template-matching method [69,70], which identifies synaptic events based on shape matching to a data-based ideal template. In *Drosophila *embryos and L1 larvae, small sEJCs as recorded and analyzed for this study represent a mixture of spontaneous calcium-dependent and calcium-independent monovesicular events. Changing the calcium concentration to 0 mM in the recording saline decreases the frequency of embryonic/L1 sEJCs, but not their amplitude [68].

### Statistics

All statistical comparisons were made using unpaired T-tests or, for distributions, Kolmogorov-Smirnov tests. Statistical significance in figures is represented as follows: * = p < 0.05, ** = p < 0.01, and *** = p < 0.001. All error bars represent S.E.M.

## Authors' contributions

Faith Liebl performed the genetics and immunocytochemical analyses. Kaiyun Chen performed the first instar patch clamp electrophysiology and analysis. Julie Karr performed the real-time RT-PCR. Qi Sheng assisted with the screen that identified *sec8P1*, helped clone the cDNA, and created the *UAS-sec8 *transgene. David Featherstone oversaw the project, performed the immunocytochemistry shown in Figures 2A and 7A-B and is responsible for the final version of this manuscript.
